# Optimal Utility of H-Reflex RDD as a Biomarker of Spinal Disinhibition in Painful and Painless Diabetic Neuropathy

**DOI:** 10.3390/diagnostics11071247

**Published:** 2021-07-12

**Authors:** Anne Worthington, Alise Kalteniece, Maryam Ferdousi, Luca D’Onofrio, Shaishav Dhage, Shazli Azmi, Clare Adamson, Shaheen Hamdy, Rayaz A. Malik, Nigel A. Calcutt, Andrew G. Marshall

**Affiliations:** 1 Division of Diabetes, Endocrinology and Gastroenterology, Faculty of Biology, Medicine and Health, University of Manchester, Manchester M13 9PL, UK; anne.worthington@postgrad.manchester.ac.uk (A.W.); Shaheen.Hamdy@manchester.ac.uk (S.H.); 2 Division of Cardiovascular Sciences, Faculty of Biology, Medicine and Health, University of Manchester, Manchester M13 9PL, UK; alise.kalteniece@manchester.ac.uk (A.K.); maryam.ferdousi@manchester.ac.uk (M.F.); Shaishav.Dhage@christie.nhs.uk (S.D.); shazli.azmi@manchester.ac.uk (S.A.); ram2045@qatar-med.cornell.edu (R.A.M.); 3 Department of Experimental Medicine, Sapienza University, 00185 Rome, Italy; luca.donofrio@uniroma1.it; 4 Diabetes Centre, Manchester University NHS Foundation Trust, Manchester M13 0JE, UK; clare.adamson@mft.nhs.uk; 5 Weill Cornell Medicine-Qatar, Research Division, Qatar Foundation, Education City, Doha 24144, Qatar; 6 Department of Pathology, University of California, San Diego, CA 92093-0612, USA; ncalcutt@health.ucsd.edu; 7 Division of Neuroscience and Experimental Psychology, Faculty of Medical and Human Sciences, University of Manchester, Manchester M13 9PL, UK; 8 Institute of Life Course and Medical Sciences, University of Liverpool, Liverpool L69 3BX, UK

**Keywords:** diabetic neuropathy, pain, spinal disinhibition, H-reflex

## Abstract

Impaired rate-dependent depression of the Hoffman reflex (HRDD) is a potential biomarker of impaired spinal inhibition in patients with painful diabetic neuropathy. However, the optimum stimulus-response parameters that identify patients with spinal disinhibition are currently unknown. We systematically compared HRDD, performed using trains of 10 stimuli at five stimulation frequencies (0.3, 0.5, 1, 2 and 3 Hz), in 42 subjects with painful and 62 subjects with painless diabetic neuropathy with comparable neuropathy severity, and 34 healthy controls. HRDD was calculated using individual and mean responses compared to the initial response. At stimulation frequencies of 1, 2 and 3 Hz, HRDD was significantly impaired in patients with painful diabetic neuropathy compared to patients with painless diabetic neuropathy for all parameters and for most parameters when compared to healthy controls. HRDD was significantly enhanced in patients with painless diabetic neuropathy compared to controls for responses towards the end of the 1 Hz stimulation train. Receiver operating characteristic curve analysis in patients with and without pain showed that the area under the curve was greatest for response averages of stimuli 2–4 and 2–5 at 1 Hz, AUC = 0.84 (95%CI 0.76–0.92). Trains of 5 stimuli delivered at 1 Hz can segregate patients with painful diabetic neuropathy and spinal disinhibition, whereas longer stimulus trains are required to segregate patients with painless diabetic neuropathy and enhanced spinal inhibition.

## 1. Introduction 

There is increasing recognition that processes within the spinal cord are key to modulating neuropathic pain, through amplification or suppression of peripheral nociceptive inputs. The neurotransmitters γ-aminobutyric acid (GABA) and glycine play a critical inhibitory role in regulating spinal nociceptive processing [1,2,3]. Diabetic rodents exhibiting behavioural markers of pain paradoxically show elevated basal and stimulus-evoked levels of the inhibitory neurotransmitter GABA in spinal CSF [4,5] and a reversal of GABA_A_ receptor function, from inhibitory to excitatory, has been demonstrated [4]. Current evidence indicates a reduction in potassium (K+)/chloride (Cl−) symporter KCC2 in the dorsal spinal cord and subsequent elevation in intracellular/intraneural chloride concentration, which renders GABA, acting via GABA_A_ receptors, less hyperpolarising and potentially depolarising [4]. This pro-nociceptive alteration is termed spinal disinhibition [6]. 

A potential biomarker of spinal disinhibition is rate-dependent depression of the H-reflex [7,8]. The H-reflex enables the study of proximal peripheral nerve conduction and provides insight into the function of spinal modulatory systems [9]. The H-reflex is modified when exposed to successive stimulations resulting in a decrease in the H-wave amplitude relative to the amplitude of the original H-wave [10] and is termed rate-dependent depression (HRDD). Although the precise neurophysiological substrate remains unclear, and indeed may be multifactorial, the magnitude of HRDD is known to be dependent on the function of inhibitory mechanisms in the spinal cord [11,12,13]. Accordingly, a loss of inhibition that accompanies the spasticity seen in animal models of spinal cord injury and following spinal cord injury in humans, results in a reduction in the magnitude of HRDD [10,14]. 

Both a loss of HRDD and altered behavioural indices of neuropathic pain are seen in rodent models of diabetes [4]. Moreover, both are also associated with reversal of GABA_A_ inhibition, suggesting that they share a common pathogenic mechanism; spinal disinhibition [4,7]. Our recent research has demonstrated that loss of HRDD occurs in patients with painful diabetic neuropathy [8,15]. We suggest that loss or attenuation of HRDD may serve as a marker of spinal inhibitory dysfunction in a sub-set of patients where spinal disinhibition contributes to neuropathic pain. Thus, a potential clinical application of HRDD could be to enable identification of patients with painful diabetic neuropathy in whom the pain has either dominant peripheral or spinal drive, to allow mechanistically informed clinical trials and predict response to specific anti-neuropathic pain medications.

Currently, it is not known which HRDD parameters best distinguish between these sub-groups of patients. In preclinical and clinical studies, the stimulation trains used for eliciting HRDD have been of variable length and frequency, and HRDD has been calculated using a variety of individual or average response sequences. In our proof-of-concept clinical study in patients with diabetes, trains of three stimuli were delivered [8]. HRDD was calculated as the amplitude of stimulus 1 compared to stimulus 3, as qualitatively it was observed that H-reflex depression was greater at stimulus 3 than stimulus 2 [8]. Stimulation at 1 Hz produced the greatest separation between patients with and without painful neuropathy. In a follow-up study of a larger cohort of patients with diabetic neuropathy [15], trains of 10 stimuli were delivered at 1 Hz and the response to the initial stimulus was compared to the mean of all subsequent responses to mitigate against random and time course fluctuations. 

Using signal detection theory methods, the purpose of the current investigation was to systematically assess which stimulation and response parameters best distinguish between patients with and without pain. This is critical to allow wider application of this technique as a balance needs to be struck between obtaining the most useful and relevant information with the practicalities of performing and tolerability of the investigation. To explore the utility of HRDD as a biomarker in painful diabetic neuropathy, we compared response trains at increasing stimulus frequencies, with the aim of defining the optimal parameters to segregate patients with spinal inhibitory dysfunction as a dominant pain mechanism. 

## 2. Materials and Methods

### 2.1. Ethics and Recruitment

Patients were recruited from secondary care diabetes clinics at one NHS trust over a period of 24 months. Convenience sampling was used, and all eligible patients were given the opportunity to take part in the research study. Control subjects were recruited via hospital and University advertisements. Written informed consent was obtained for each participant. Study conduct adhered to the tenets of the Declaration of Helsinki and Research Ethics Committee approval was granted on 28 February 2017 (Project identification code: 220627, East Midlands, Leicester South Research Ethics Committee, reference 17/EM/0076). 

### 2.2. Participants and Demographics

Patients with painful diabetic neuropathy (*n* = 53), patients without painful diabetic neuropathy (*n* = 75) and healthy control subjects (*n* = 36) were studied. The H-reflex was absent in 24 patients with diabetes (10 with type 1 and 14 with type 2 diabetes) and 2 control subjects, and these subjects were excluded from further assessment. Detailed demographic data, medical history and current medications, age, gender, ethnicity, type and duration of diabetes, co-morbidities, height, weight, blood pressure, HbA1c, lipids and renal function were documented. Other common causes of neuropathy were excluded based on a family history as well as testing for serum B_12_, folate, immunoglobulins, electrophoresis and anti-nuclear antibody. Participants underwent assessment of small and large nerve fibres using corneal confocal microscopy, nerve conduction studies and thermal thresholds along with assessment of HRDD. Visual analogue scale (VAS) pain scores (0–100) recording current, average and maximum pain ratings over the previous 24 h were documented. Patients were stratified into painful (DPN+) and painless (DPN-) cohorts based on the Toronto consensus that “the symptoms are distal, symmetrical, often associated with nocturnal exacerbations, and commonly described as prickling, deep aching, sharp, like an electric shock, and burning with hyperalgesia” for greater than 3 months, consistent with the requirement of pain chronicity defined by the International Association for the Study of Pain (IASP) [16]. All patients with a current, average or maximum VAS pain score >0 were placed in the pain cohort.

### 2.3. Nerve Conduction and H-Reflex Studies

**Figure 1:**
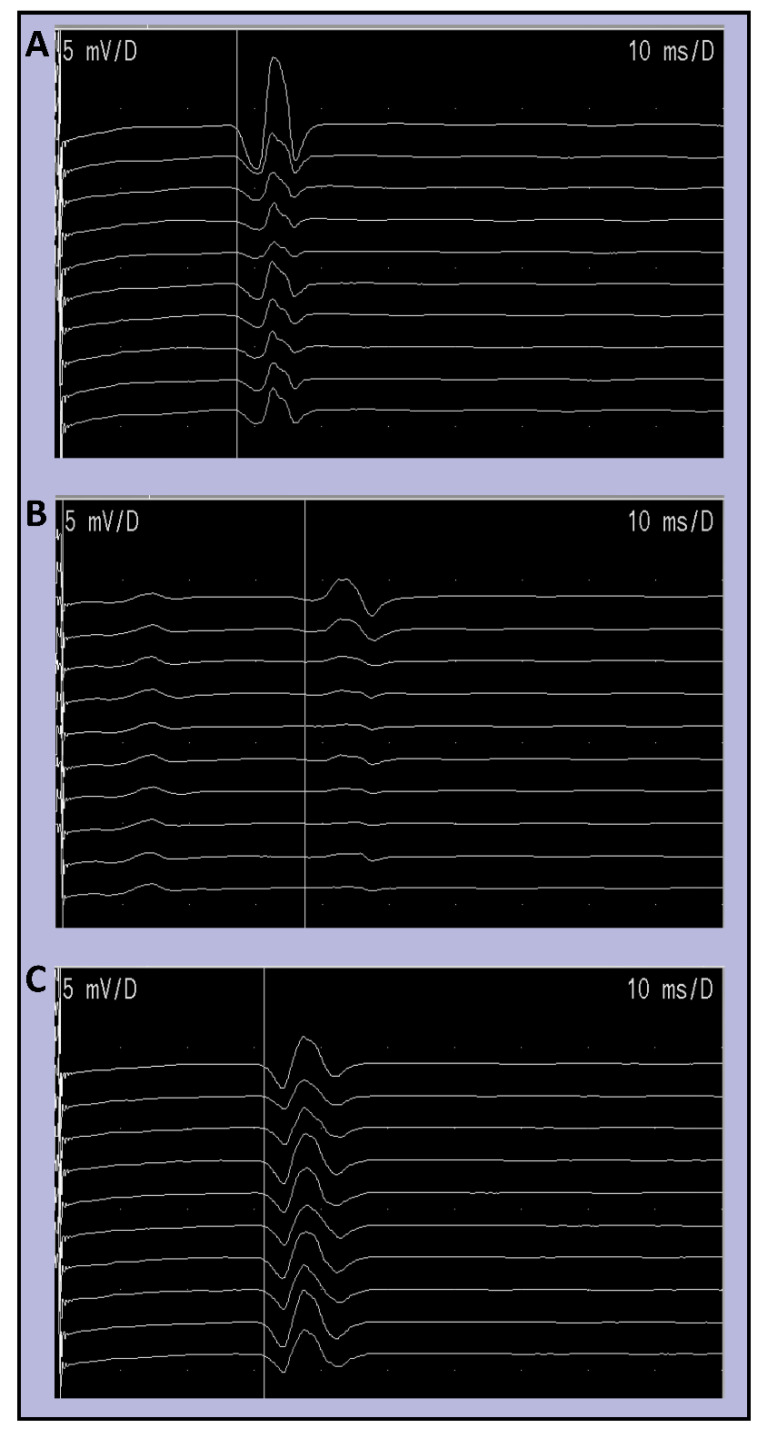
Example raw traces of H-reflex RDD in (**A**) healthy controls, (**B**) patients with diabetes without neuropathic pain, and (**C**) patients with diabetes with neuropathic pain.

Nerve conduction and H-reflex studies were performed using a DANTEC Keypoint system (Dantec Dynamics Ltd., Bristol, UK). Participants were sat semi-recumbent at 45° with limb temperature maintained between 32–35°. Sural sensory amplitude (SNAP) and conduction velocity (SNCV) along with peroneal motor nerve amplitude (PMNAP) and conduction velocity (PMNCV) were recorded. For H-reflex studies, tibial nerve stimulation was performed using 1 ms square wave monophasic pulses delivered using surface silver-silver chloride electrodes to the popliteal fossa. Surface silver-silver chloride recording electrodes with a diameter of 9 mm were placed on the long axis of soleus. H-reflex recruitment curves were obtained to determine peak-peak H-reflex maximal amplitude (Hmax) by incrementing stimulation current by 1 mA. A minimal stimulation interval of 10 s was observed. For HRDD, a submaximal stimulus strength (to achieve a response of 75% of max) was used. A train of 10 stimuli were delivered (Figure 1) at 0.3, 0.5, 1, 2 and 3 Hz, giving 10 H-responses termed H1–H10 respectively. The frequency of the stimulation trains were delivered in a pseudo-randomised order with a minimum inter-train interval of 10 s. HRDD was calculated as the individual response value expressed as a percentage of response H1 or the mean of a group of responses, expressed as a percentage of response H1. 

### 2.4. Corneal Confocal Microscopy

Participants underwent corneal confocal microscopy (CCM) using a laser scanning corneal confocal microscope HRT III (Heidelberg Retinal Tomograph III Rostock Cornea Module, Heidelberg Engineering, Heidelberg, Germany) for both eyes using an established protocol [17]. Six images from the central cornea at the level of the sub basal nerve plexus were analysed for each patient and control subject. Corneal nerve fibre density (CNFD, total number of main nerves per square millimetre (no./mm^2^)), corneal nerve fibre length (CNFL, total length of main nerves and nerve branches per square millimetre) (mm/mm^2^) and corneal nerve branch density (CNBD, total number of nerves per square millimetre (no./mm^2^)) were quantified. 

### 2.5. Statistical Methods

Statistical analyses were performed using GraphPad Prism statistical software (GraphPad Software Inc, La Jolla, CA, USA). Parametric data were analysed using unpaired t-test or one-way ANOVA followed by Tukey’s multiple comparison to compare means between groups. Non-parametric data were analysed using the Kruskal–Wallis test followed by Dunn’s post hoc for multiple comparisons. The analysis of covariance (ANCOVA) (post hoc least significant difference (LSD)) was used to compare variables between groups, while statistically controlling for the effects of age. Receiver operating characteristic (ROC) curve analyses were used to define the Wilson/Brown estimate of area under the ROC curve. Optimal cut offs were determined using Youden’s index [18] and associated sensitivity and specificity documented. 

## 3. Results

### 3.1. Clinical, Demographic and Neuropathy Measures

Clinical and demographic characteristics are summarised in Table 1. In the painful diabetic neuropathy group, 16 patients were taking anti-neuropathic pain medication as monotherapy (six were taking selective serotonin reuptake inhibitors, four were taking tricyclic medication, three were taking gabapentanoids and three were taking Duloxetine). Patients with diabetes were older, with higher BMI and HbA1c compared to control subjects. There was no significant difference in BMI or HbA1c between patients with or without neuropathic pain. Sural amplitude, sural and peroneal conduction velocity, corneal nerve fibre density and corneal nerve fibre length were all significantly lower in patients with diabetes compared to controls. Cold detection and warm detection thresholds were significantly impaired in patients with diabetes compared to controls. There was no significant difference between patients with and without painful neuropathy in any of the demographic parameters or measures of small and large fibre neuropathy. 

### 3.2. HRDD Time-Course

**Figure 2:**
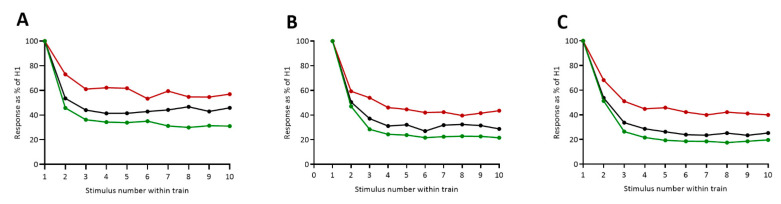
Group mean H wave amplitude responses to stimulus train at (**A**) 1 Hz, (**B**) 2 Hz and (**C**) 3 Hz in control subjects (black circle/black line), patients with painful diabetic neuropathy (red circle/red line) and patients with painless diabetic neuropathy (green circle/green line). Significance values detailed in Table 2.

To explore the time course of HRDD during stimulation trains in patients with diabetes and control subjects, analysis of individual stimulus responses and train averages was performed at five stimulation frequencies (Table 2). There was no significant difference at any stimulus number between patients with or without painful diabetic neuropathy or between control subjects and either patient group at 0.3 Hz. With increasing stimulus frequency, an increasing number of significant differences were seen. At 0.5 Hz, there were significant differences in individual stimulus responses (H2, *p* = 0.006; H3, *p* ≤ H5, *p* ≤ 0.001; H6, *p* ≤ 0.016) and train averages (mean H2–4, *p* = 0.0214; mean of H2–5, *p* < 0.003; mean of H2–10, *p* < 0.018) between patients with and without painful neuropathy. At 1, 2 and 3 Hz (Figure 2, Table 2), all individual and train averages between patients with and without painful neuropathy were significantly different (*p* < 0.001 except for H2 at both 2 Hz (*p* < 0.034) and 3 Hz (*p* < 0.001)). There was a significant difference between patients with painful neuropathy and control subjects at 1Hz (H2, H3, H4, H5 and H7, all *p* < 0.01), 2 Hz (H3, H4, H5, H6 and H10, all *p* < 0.05) and 3 Hz (stimulation numbers H2–10, all *p* < 0.05). A significant difference was observed between patients without painful neuropathy and controls for H2 at 0.5 Hz (*p* = 0.037) and at individual stimulus responses towards the end of the train (H7, *p* = 0.012; H8, *p* = 0.004; H10, *p* = 0.003) and whole train average (mean of H2–10, *p* = 0.018) at 1 Hz. No significant difference was seen between patients without painful neuropathy and control subjects at 2 or 3 Hz. 

### 3.3. ROC Curve Analysis

**Figure 3:**
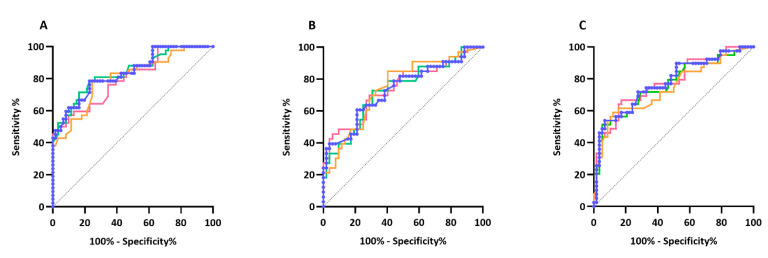
Receiver-operated characteristic (ROC) plots for HRDD at (**A**) 1 Hz, (**B**) 2 Hz, (**C**) 3 Hz in patients with painful diabetic neuropathy versus patients with painless diabetic neuropathy using stimulus response H3 (orange line), average of stimulus responses H2–4 (green line), average of stimulus responses H2–5 (blue line) and average of stimulus responses H2–10 (pink line).

To determine the optimal cut-off for HRDD between patients with and without pain, a ROC curve analysis was undertaken for both individual stimulus responses and average stimulus responses at 1, 2 and 3 Hz (Figure 3). The Wilson/Brown area under the curve was greatest for stimulus response averages H2–4 and H2–5 at 1 Hz, AUC = 0.84 (95%CI 0.76–0.92) with an optimal cut-off at 64.6 and 65.1 respectively. Increased sensitivity was observed using stimulus response average H2–4 (52.4 compared to 47.6 using stimuli 2–5) but greater specificity was seen using stimulus response average H2–5 (98.4 compared to 96.7 using stimuli 2–4) (Table 3). Analysis incorporating later responses did not improve AUC (1 Hz: H2–6, AUC = 0.82; H2–7, AUC = 0.82; H2–8, AUC = 0.81; H2–9, AUC = 0.80; H2–10, AUC = 0.80).

## 4. Discussion

We have previously demonstrated attenuated or enhanced HRDD in patients with diabetes and painful or non-painful neuropathy, respectively [8,15]. In the present study, we have defined HRDD stimulation and response parameters that best segregate patients with painful or non-painful diabetic neuropathy and determined optimal sensitivity and specificity for HRDD to stratify patients based on the mechanistic basis of their pain.

HRDD refers to the degree of suppression of the H-reflex by repetitive stimulation and increases in magnitude with increasing stimulation frequency [10]. In addition to this phenomenon, we have demonstrated an increasing divergence of H-reflex responses in patients with and without painful neuropathy at increasing stimulation frequencies. At a frequency of 1 Hz, all individual stimulus responses throughout the train were significantly different between patients with diabetes, with and without pain. The higher frequencies of 2 and 3 Hz showed equally significant differentiation but did not add in terms of distinguishing those with pain and no pain. The ROC analysis of full train averages alongside individual response H3, and mean averages of responses H2–4 and H2–5 yielded excellent discrimination [19] between patients with and without pain. In terms of patient tolerability of the assessment, stimulation at 1 Hz is rarely uncomfortable and is more acceptable than stimulation at higher frequencies and thus less likely to produce response artifacts due to contraction of other lower limb muscles [20]. We have shown that optimal discrimination between painful and painless diabetic polyneuropathy can be achieved whilst abbreviating the stimulus train to five impulses.

In the current study, patients with diabetes were segregated based on the presence and duration of characteristic neuropathic symptoms into those with pain versus those with no pain [21,22]. It should be emphasised that the primary purpose was not to define HRDD as a biomarker of pain per se, but rather as a biomarker of a pain mechanism, namely spinal disinhibition. As loss or attenuation of HRDD is indicative of abnormal spinal inhibitory processing, identification of an optimal cut off value of HRDD which best delineates patients with disinhibition as a dominant pain mechanism is paramount. In our ROC analysis, using the best discriminatory area under the curve value, mean of responses H2–4 and H2–5 at 1 Hz, followed by Youden’s index, we obtained a sensitivity value of 78.57 (confidence interval 63.19–89.70) and 78.57 (confidence 64.06–88.29), respectively. A similar value, 77.05, was also obtained for specificity. It is, however, unlikely that this balance provides the best interpretation of the data for our potential biomarker. Our previously published work [8,15] provides evidence that impaired HRDD demonstrates a degree of heterogeneity across a cohort of patients with pain, i.e., not all patients with pain have impaired HRDD. However, impaired HRDD is a highly unique feature to patients with pain, i.e., no patients without pain fell in the sub-group of patients with highly impaired (>2SD) HRDD. Therefore, an increased level of specificity (and in turn a reduced level of sensitivity) may represent a more appropriate cut off value for HRDD. For example, at 100% specificity, HRDD (using mean H2–5 @ 1 Hz) above 66.3 would capture 42.9% of patients with pain.

Our working hypothesis is that patients identified using this cut off may constitute a sub-group of patients in whom treatment with spinally acting medication is likely to be more effective. In our previous work in rodent models of diabetes, spinally administered Duloxetine, a serotonin-noradrenaline reuptake inhibitor (SNRI) thought to act by enhancing spinal inhibition via 5-HT_2A_ receptors [23], was shown to reverse both allodynia and deficits in HRDD [8]. Indeed, in clinical studies, Duloxetine is effective in treating painful diabetic neuropathy in a similar proportion of patients identified by our ROC analysis using the example of 100% specificity [24,25,26]. One could speculate that the patients identified in this way might represent those who are most likely to benefit from SNRI-based pharmacotherapy. 

Whilst it may result in a proportion of patients with spinal disinhibition being missed, selecting a high specificity value—and thus a high likelihood of only including patients in whom impaired spinal inhibitory processes are a primary pain mechanism—would be beneficial for enriching clinical trial populations, assessing mechanism-based treatment of painful diabetic neuropathy, and as a component of studies involving deep phenotyping of pain [27,28]. For example, mechanistic studies assessing putative peripheral and central mechanisms of pain generation in combination with spinal fMRI may ultimately pave the way towards personalised pain medicine. To achieve this, further biomarker refinement will be required to segregate patients that respond to particular drugs such as Duloxetine or Gabapentin, such that HRDD might inform physicians about the likely efficacy of spinally acting drugs.

Whilst shorter stimulus trains are appropriate, and likely to be more clinically acceptable, for detecting impairment in HRDD in painful diabetic neuropathy, they may miss the enhancement of HRDD seen in patients with diabetes who have no pain. Our recent study suggested that HRDD, and by implication spinal inhibition and pain suppression, may be enhanced in patients with painless diabetic neuropathy [15]. In the current study the only stimulation frequency which differentiated patients with diabetic neuropathy without pain and control subjects was 1 Hz. It is of note that this was only observed in responses towards the end of the stimulus train; post 6 s. Furthermore, maximal depression of the H-reflex was reached progressively later throughout the response train, with increasing stimulus frequency. This may suggest that the degree of HRDD is dependent on time, in addition to the well-documented dependence on stimulus frequency. These findings may also indicate that, unlike the impairment in HRDD in painful diabetic neuropathy, which is evident throughout the stimulus train, longer stimulus trains are likely to be needed to capture these potential later pain suppressive differences in spinal inhibitory function seen in painless diabetic neuropathy. Recent studies in the streptozotocin (STZ) rat model of type I diabetes suggest that the development of spinal disinhibition in diabetes is a time-dependent process involving both GABA_A_- and GABA_B_-mediated responses [29]. Initially deficits in HRDD are not seen because GABA_B_ mediated inhibition appears to compensate for the reversal in polarity of GABA_A_. GABA_B_ receptor-mediated hyperpolarisation, in contrast to the fast inhibitory signalling mediated by GABA_A_ receptors, is slow and prolonged [30]. Therefore, one possibility is that in patients with painless diabetic polyneuropathy, the enhancement of HRDD and spinal inhibition is due to a compensatory GABA_B_-mediated hyperpolarisation that takes time to develop during the stimulus train. 

Potential limitations of the study include the relatively small cohort of patients and the fact that a proportion of patients in the painful diabetic neuropathy group were taking anti-neuropathic pain medication. Furthermore, patients were taking a variety of different anti-neuropathic pain medications (see results) with diverse mechanisms of action, precluding a systematic assessment of their effects on HRDD. It is currently not known whether anti-neuropathic pain medication affects HRDD. One further potential limitation of the study is that the control group was of significantly lower age than both the painful and painless diabetic neuropathy groups. It should be recognised, however, that our previous studies showed no significant correlation between HRDD and age. It also does not affect the ROC analysis as data from control participants was not used for this analysis. 

## 5. Conclusions

The current study provides a detailed comparison between individual and average HRDD stimulus responses to distinguish patients with painful diabetic neuropathy, specifically those whose pain is likely driven by spinal disinhibition. An average of responses H2–5 provided good to excellent predictive ability at all three frequencies, with 1 Hz proving optimal. In terms of clinical utility, the shortened train of five stimuli combined with a moderate stimulation frequency of 1 Hz makes HRDD a valuable tool which can easily be accommodated in a clinical setting in terms of equipment, time and most importantly patient tolerability. Longer trains may be required to investigate enhanced inhibitory processing in the spinal cord in painless diabetic neuropathy.

## Figures and Tables

**Table 1:** Demographic and neuropathy parameters for patients with diabetes, with and without neuropathic pain, and control subjects.

	Diabetes with Neuropathic Pain (*n* = 42)	Diabetes without Neuropathic Pain (*n* = 62)	Controls (*n* = 34)
Type of Diabetes (1/2)	11/31	26/36	
Gender (Female/Male)	18/24	18/44	20/14
Ethnicity (White/Asian/Black)	32/8/2	46/13/3	25/7/2
	Median ± Interquartile Range		
Age (years)	61.5 (49.8–69.5) ***	65.0 (51.5–71) ****	46.5 (31–55)
Duration (years)	12.5 (4.8–20.3)	16.0 (10.0–23.3)	
HbA1c %(mmol/mol)	7.0 (6.2–7.5) *** 53.0 (44.5–58) ***	7.5 (6.8–8.4) **** 58.5 (51–68.25) ****	5.4 (4.9–5.7) 35.0 (30.5–38.75)
BMI (kg/m²)	29.2 (25.5–34.9) ****	27.3 (24.3–31.9) **	23.8 (22.5–25.4)
SNAP (µV)	7.7 (3.7–15) ***	6.9 (4.3–11.5) ****	17.0 (15–22)
SNCV (m/s)	43.1 (40–46.7) ***	42.4 (40–46.7) ****	48.3 (45.2–51.9)
PMNAP (mV)	3.8 (2.4–5.7)	3.5 (2.4–5.9) *	4.9 (3.4–7.5)
PMNCV (m/s)	41.4 (38.1–43.7) ****	41.2 (38.6–44) ****	47.5 (43.4–50)
CDT (°C)	27.8 (23.3–29.6) ***	28.0 (24.1–29.9) **	29.8 (28.5–30.5)
WDT (°C)	40.3 (36.4–46.3) **	40.0 (37.7–43.6) ***	36.3 (34.8–39.3)
VAS Pain Current (0–100)	20 (8–46.5)		
VAS Pain Average past 24 h (0–100)	35.5 (15.8–64.5)		
VAS Pain Maximum past 24 h (0–100)	56 (30–80)		
	Mean ± SE		
CNFD (no.mm²) ^	23.84 ± 1.84 ***	25.30 ± 1.31 ***	32.36 ± 1.59
CNFL (mm/mm²) ^	18.16 ± 1.563 ***	18.43 ± 1.11 ***	25.11 ± 1.35
CNBD (no.mm²) ^	46.64 ± 5.18	47.61 ± 4.20	59.74 ± 6.85
HRDD meanH2-10 @ 1 Hz ^	60.40 ± 2.74 ***	34.92 ± 2.33 * +++	43.46 ± 3.31

Non-parametric data are expressed as median ± interquartile range: Mann–Whitney or Kruskal–Wallis with Dunn post hoc test. Parametric data are expressed as mean ± SE: ^ ANCOVA values adjusted for age (post hoc LSD). * *p* < 0.05 compared to controls ** *p* < 0.01 compared to controls *** *p* < 0.001 compared to controls **** *p* < 0.0001 compared to controls. +++ *p* < 0.001 compared to painful diabetic neuropathy. BMI, body mass index; SNAP, sural nerve amplitude; SNCV, sural nerve conduction velocity; PMNAP, peroneal motor nerve amplitude; PMNCV, peroneal motor nerve conduction velocity; CDT, cold detection threshold; WDT, warm detection threshold; VAS, visual analogue scale; CNFD, corneal nerve fibre density; CNFL, corneal nerve fibre length; CNBD, corneal nerve branch density; HRDD, H-reflex rate-dependent depression.

**Table 2:** Significance values for individual stimulus responses and train averages between patients with diabetes, with and without pain, and control subjects at 0.3, 0.5, 1, 2 and 3 Hz. Between group means compared using one-way ANOVA and Tukey’s post hoc multiple comparison. Data are expressed as *p* value determined by Tukey’s post hoc test.

		H2	H3	H4	H5	H6	H7	H8	H9	H10	Average of H2–4	Average of H2–5	Average of H2–10
0.3 Hz	Pain v No Pain	0.177	0.552	0.551	0.417	0.366	0.273	0.906	0.077	0.177	0.279	0.272	0.229
	Pain v Control	0.868	0.673	0.955	0.940	0.692	0.960	0.906	0.786	0.941	0.793	0.944	0.979
	No Pain v Control	0.063	0.134	0.388	0.677	0.916	0.467	0.998	0.359	0.351	0.078	0.161	0.363
0.5 Hz	Pain v No Pain	0.006	0.004	0.621	0.001	0.016	0.498	0.382	0.053	0.248	0.021	0.003	0.018
	Pain v Control	0.837	0.591	0.839	0.066	0.255	0.996	0.928	0.772	0.680	0.951	0.546	0.655
	No Pain v Control	0.037	0.074	0.262	0.356	0.541	0.434	0.191	0.245	0.763	0.051	0.075	0.175
1 Hz	Pain v No Pain	<0.0001	<0.0001	<0.0001	<0.0001	<0.0001	<0.0001	<0.0001	<0.0001	<0.0001	<0.0001	<0.0001	<0.0001
	Pain v Control	<0.001	<0.001	<0.001	<0.001	0.079	0.006	0.310	0.080	0.057	<0.0001	<0.0001	<0.001
	No Pain v Control	0.236	0.147	0.256	0.233	0.183	0.012	0.004	0.055	0.003	0.125	0.117	0.018
2 Hz	Pain v No Pain	0.034	<0.001	<0.0001	<0.0001	<0.0001	<0.0001	<0.001	<0.001	<0.001	<0.0001	<0.0001	<0.0001
	Pain v Control	0.236	0.031	0.006	0.050	0.009	0.103	0.317	0.108	0.039	0.016	0.014	0.018
	No Pain v Control	0.753	0.320	0.275	0.185	0.462	0.107	0.093	0.125	0.386	0.327	0.254	0.175
3 Hz	Pain v No Pain	<0.001	<0.0001	<0.0001	<0.0001	<0.0001	<0.0001	<0.0001	<0.0001	<0.0001	<0.0001	<0.0001	<0.0001
	Pain v Control	0.022	0.005	0.007	<0.001	0.001	0.002	0.009	0.002	0.016	0.002	<0.001	<0.001
	No Pain v Control	0.867	0.320	0.304	0.259	0.627	0.482	0.306	0.573	0.482	0.393	0.342	0.397

**Table 3:** Receiver-operated characteristic (ROC) analysis with area under the curve, optimal cut off and respective sensitivity and specificity with 95% confidence interval, for H-reflex stimuli responses at 1, 2 and 3 Hz in patients with painful and painless diabetic neuropathy. CI, confidence interval.

	Optimal Cut Off	Area under Curve 95% CI	Sensitivity	Specificity
			95% CI	95% CI
**1 Hz H3**	45.73	0.8	78.57	75.42
		0.72–0.89	63.19–89.70	62.71–85.54
**1 Hz mean H2–3**	58.93	0.82	88.52	64.29
		0.73–0.90	78.16–94.33	49.17–77.01
**1 Hz mean H2–4**	49.65	0.84	78.57	77.05
		0.76–0.92	63.19–89.70	64.50–86.85
**1 Hz mean H2–5**	49.13	0.84	78.57	77.05
		0.76–0.92	64.06–88.29	65.09–85.81
**1 Hz mean H2–10**	57.49	0.8	50.34	96.72
		0.72–0.89	34.19–65.81	88.65–99.60
**2 Hz H3**	29.81	0.74	84.85	59.62
		0.64–0.85	69.04–93.35	46.07–71.84
**2 Hz mean H2–3**	37.66	0.7	53.85	81.82
		0.58–0.81	40.5–66.66	65.61–91.39
**2 Hz mean H2–4**	38.84	0.73	72.73	69.23
		0.62–0.84	55.78–84.93	55.73–80.09
**2 Hz mean H2–5**	42.84	0.73	60.61	78.85
		0.62–0.85	43.68–75.32	65.97–87.76
**2 Hz mean H2–10**	33.61	0.74	69.7	71.15
		0.63–0.85	57.73–81.67	57.73–81.67
**3 Hz H3**	48.49	0.74	56.41	89.66
		0.64–0.85	40.98–70.70	79.21–95.17
**3 Hz mean H2–3**	43.54	0.74	63.79	79.49
		0.64–0.85	50.93–74.95	64.47–89.22
**3 Hz mean H2–4**	57.12	0.76	51.28	94.83
		0.66–0.86	36.20–68.13	85.86–98.59
**3 Hz mean H2–5**	50.91	0.78	53.85	93.1
		0.68–0.87	38.57–68.43	83.57–97.29
**3 Hz mean H2–10**	F	0.78	66.67	82.76
		0.68–0.87	50.98–79.37	71.09–90.36

## Data Availability

The data presented in this study are available on request from the corresponding author.
